# MULTI-COMPONENT RELAXOMETRY FOR STUDY OF AGING

**DOI:** 10.1093/geroni/igad104.1278

**Published:** 2023-12-21

**Authors:** Shannon Kolind

**Affiliations:** University of British Columbia, Vancouver, British Columbia, Canada

## Abstract

While relaxation characteristics of MRI are used regularly for conventional, qualitative imaging, more information can be extracted by fitting multiple water components to the measured signal. Parameters including the myelin water fraction or T2 of intra/extracellular water can provide information about myelin development and degeneration, or inflammation and edema. Multiple approaches to both acquisition and analysis have emerged. This talk will provide an overview of some recent advancements and uses in aging.

